# Applications of High Content Screening in Life Science Research

**DOI:** 10.2174/138620709789383277

**Published:** 2009-11

**Authors:** Joseph M Zock

**Affiliations:** Thermo Fisher Scientific, USA

**Keywords:** High content screening, cell signaling, oncology, neurobiology, in vitro toxicology, target identification, target validation, RNAi, stem cells.

## Abstract

Over the last decade, imaging as a detection mode for cell based assays has opened a new world of opportunities to measure “phenotypic endpoints” in both current and developing biological models. These “high content” methods combine multiple measurements of cell physiology, whether it comes from sub-cellular compartments, multicellular structures, or model organisms. The resulting multifaceted data can be used to derive new insights into complex phenomena from cell differentiation to compound pharmacology and toxicity. Exploring the major application areas through review of the growing compendium of literature provides evidence that this technology is having a tangible impact on drug discovery and the life sciences.

## INTRODUCTION

High Content Screening (HCS) is the application of automated microscopy and image analysis to both drug discovery and cell biology. This technique has grown from an interesting proposition, to a useful technology, and onto a valuable utility over the last decade. This paper reports on observations of peer reviewed journal articles using HCS as a key component of the research and attempts to offer a glimpse at how widely adopted the technology has been in several important areas of life science research.

Predictably, early papers were focused on HCS as a novel technology. As the technology has gained wider acceptance and use, however, the focus of papers has shifted back to the biology being studied, with HCS becoming one of the tools to deliver “supportive biological context” to whatever new entity or idea is being proposed. In my mind, this trend is one of the hallmarks of true technology adoption and a good indication that HCS is here to stay.

It is interesting to note that, although most of scientific articles citing the use of HCS fall along the categories of cell signaling, oncology, neurobiology, *in vitro* toxicology, and target validation (i.e. RNAi), an increasing number of papers describe very novel applications. Adoption beyond the now “standard” uses for HCS shows the flexibility of the technology to expand the breadth of addressable biological processes including cardiac failure [1], gap junctions [2], immunosupression [3], osteoporosis [4], phagocytosis [5], autophagy [6], centrosome function [7], fungal pathogenesis [8], retinal repair [9], circadian rhythms [10], and screening in yeast [11] just to name a few.

As with any emerging technology, HCS is being compared to current assay methods. In many instances HCS provides significant benefits over existing approaches or at least is seen as complementary, providing additional data that can be used to make scientific conclusions. In recent papers comparing HCS approaches to a standard Enzyme - Linked ImmunoSorbent Assay (ELISA), for example, it is noted that the ability to see what is going on at individual cell resolution makes the measurements more accurate and reliable [12, 13].

Liu *et al*., explain how ELISA and HCS provide synergistic outputs that help determine the complex pharmacology of the compounds they are evaluating. “It is clear that the readout from the Neurofilament (NF) ELISA is a measure of neuronal survival…on the other hand, the Cellomics ArrayScan platform, as represented in this study, is meant to evaluate a compound’s ability to increase total neurite outgrowth…the utility of both assay platforms would enable the investigator to identify compounds with multiple cellular activities, such as FK506” [14].

Agler *et al*., cite subcellular location of protein/protein interaction as an advantage of HCS over second signal assays. The ability of HCS to identify only a defined subset of cells in the well enables transient transfection to be used for robust screening instead of the forced use of “stable clones” where over-expression can lead to aberrant physiology and toxicity. “HCS assays provide other information unavailable from fluorescence polarization (FP) and reporter assays, such as subcellular localization where protein-protein interactions occur. Within this assay triage strategy, the HCS translocation assay provides an inexpensive assay format compared to purchasing commercially available reagents for FP and reporter assays” [15].

HCS used to replace difficult assays is on the rise where the advantage might be sensitivity over existing methods, increased throughput, increased safety, and/or decreased cost. Baniecki *et al*., who developed HCS to identify new anti-malarial drugs, state, “we are able to detect as little as one individual parasite in our image-based DAPI *P. falciparum* growth assay compared to a uniform well readout of 0.25% parasites observed in the DAPI *P. falciparum* growth assay and [^3^H]hypoxanthine assay - significantly greater sensitivity and reliability” [16]. Johnson *et al*., at the Center for Disease Control (CDC), developed a rapid, high throughput vaccinia virus neutralization assay [17] utilizing HCS to replace assays that are “laborious, particularly for large numbers of test sample and take up to 48-72 hours for plaque formation…analysis of results takes additional time and may be subjective since the plaques are counted manually”. The HCS assay, based on detecting viral infection with GFP, “has the potential to replace plaque reduction neutralization titer (PRNT) as a lab-standard clinical sample neutralization assay due to the speed and reliability with which data is produced. In the event of an orthopoxvirus outbreak, the speed and high throughput nature of the assay may prove extremely valuable”.

Historically, HCS has its origins in drug discovery, initially providing novel secondary assay formats, selectivity screens, and cytotoxicity profiling using the multi-parameter and individual cell attributes of the approach. “High content, in context, and with correlation” describes the data coming from the current HCS platforms [18], but understanding the value and utility of what that data represents is only now becoming obvious in the literature. In a brilliant example, Young *et al*., elegantly show how powerful the application of multi-faceted HCS data can be in their recent paper that integrates HCS and ligand-target prediction to identify pharmacological mechanisms of action [19]. Using cell cycle permutation as the model, a series of image-based cytological features were collected. A thirty-six-feature subset was selected, defining six factors (nuclear size, replication, mitosis, nuclear morphology, EdU texture, and nuclear ellipticity). A library of 6,547 compounds was profiled. “The responses grouped active compounds into seven major categories of phenotypic effects. We then explore how phenotypic profiles of active compounds compare with chemical structure and predicted target profiles. The resulting structure-activity relationships are richer than would be possible with a single data type, and they allow us to infer mechanisms of action for some compounds.”

## HCS AND CELL SIGNALING

In the drug discovery process, understanding how environmental triggers cue a particular set of biological process cascades holds the key to therapeutic control. Cell signaling, therefore, is at the root of most target-specific attempts to create drugs. In an academic setting, there is a similar need to understand how newly discovered proteins map into and across various pathways. Therefore, it is not surprising to find that many of the peer reviewed journal articles citing the use of HCS are reporting on some aspect of cell signaling. From the initial HCS paper on NFkB translocation [20] a decade ago many signaling molecule activities have been quantitated including STAT [21, 22], wnd/fzd [23], akt [24], NFAT [25], p38 [26], TGF-beta [27] and Smad2/3 [28, 29] making up signaling networks from inflammation [30] to G-Protein coupled receptors [31-33].

## HCS AND ONCOLOGY

HCS found an initial foothold in oncology research, due to the early applications for measuring apoptosis [34-38] and proliferation [39-41]. Assays for cell cycle [42-44], transformation [45] and migration [46-48] followed. At one point we developed a motility assay algorithm and reagent kit that is used to access metastatic potential [49-51]^.^ Being able to see the individual cell responses verses a “population average” has led to a better understanding of how anti-cancer compounds might differentially effect cancerous cells compared to normal cells and has been used to determine the function of cancer biomarkers [52]. Anti-cancer compounds identified utilizing HCS have started moving into clinical trials [53].

As HCS technology continued to develop, additional applications in oncology research began to emerge. The study of angiogenesis, for example, can now be routinely performed by stimulating endothelial cells to undergo angiogenic transformation in a microplate well [54, 55]. The phenotype of tube formation is striking in the sense that a multitude of cells are signaling each other and working in unison to create an extremely specific multicellular structure that has many implications on various disease states, depending on the particular focus. Stimulating neovascularization of a damaged organ or wound would be considered beneficial while inhibiting the neovascularization of a solid tumor or retina (wet form of macular degeneration) would also be therapeutic. The key here is to be able to accurately capture, measure, and report on the phenotypes by individually assessing a number of attributes. In the case of angiogenic tube formation, being able to measure tube size and shape, connectedness, number of nodes, number of cells in a tube, and even target activation in the cells in a tube allow the researcher to discriminate between compound activities. Another area of interest in oncology using HCS is the evaluation of anti-cancer compounds through the quantitation of cytoskeletal rearrangement, specifically looking at microtubule assembly/disassembly [56, 57].

A good example of HCS adoption is the NCI Institute for Chemical Genomics. They have developed and implemented HCS assays for nuclear foci formation, cell morphology changes and protein translocations. “Because such measurements are done at the cellular level rather than averaged over a well, the signal-to-noise ratio is considerably higher; each well, in essence, serves as its own set of data points” [58]. Using HCS they have been able to identify novel cell division modulators with different modes of action than the microtubule disruption of classic antimitotic compounds, secramine, an actin polymerization inhibitor reducing metastasis, and several modulators of NFAT and FOX01a nuclear translocation.

## HCS AND NEUROBIOLOGY

Very early in the development of HCS we recognized the potential of imaging in the quantitation of neuronal morphology and were the first to create a product to monitor neurite outgrowth. Over the years we have evolved the algorithmic approaches a number of times, responding to the feedback from multiple users screening for stimulation of neurite outgrowth [59-62] and neuronal protection [63-65] with the resulting calculated feature set allowing the extraction of many attributes of neurons and neuronal sub-populations in both primary cells and standard cell lines.

More striking is the application of HCS to the study of a wide variety of neurological disease states, whether it is a basic understanding of the underlying biology, the creation of new models, or the screening of molecules for therapeutic intervention. Examples include Alzheimer Disease [66], Parkinson’s Disease [67, 68], Huntington Disease [69, 70], Amyotrophic Lateral Sclerosis [71] and brain cancer [72] with more articles appearing each year.

## HCS AND *IN VITRO* TOXICOLOGY

All HCS assays can be considered “tox” assays on some level, since they measure a cell’s physiological responses to stimulus, whether it be environmental or chemical. From relatively simple measures of acute cytotoxicity, such as cell counting and cell rounding, to more specific measures of organelle health [73, 74], HCS can be applied to many situations, often as a multi-parameter assay where cross correlation of multiple endpoints can help define subtle toxic states.

It is clear that HCS has found a strong foothold in drug discovery in the area of cytotoxicity [75]. However, uses of HCS beyond straight cytotoxicity [76], where the evaluation at the cell level is predictive for downstream toxic effects in whole organisms (like us) is an important area of growth for automated imaging in drug discovery, since the increased capacity for getting critical data at the right time can mean the gain/loss of billions of dollars. Initial applications include assays for micronucleus induction [77, 78] to assess genotoxicity, phospholipidosis [79] for liver lipidosis, and developmental neurotoxicity [80]. Moving forward there is great potential in using HCS to set up new models for toxicity [81] including the use of model organisms like zebrafish and worms.

One of the most exciting results showing the potential of a multi-parameter imaging approach to *in vitro* toxicology is in the area of drug-induced liver injury where Xu *et al*., have developed a testing strategy around a panel of phenotypes that are directly linked to hepatotoxicity [82]. “When applied to over 300 drugs and chemicals including many that caused rare and idiosyncratic liver toxicity in humans, our testing strategy has a true-positive rate of 50-60% and an exceptionally low false-positive rate of 0-5%”

In another retrospective study of hepatotoxic compounds, O’Brien *et al. *[83]. compared the “standard 7” biochemical cytotoxicity assays used in the industry to a single, 4-dye component, multi-parameter HCS assay. The HCS assay showed a much higher sensitivity (93% *vs* <25%) and specificity (98% *vs* ~90%) than the best combination of the "standard 7" biochemical assays. These studies are being confirmed across the pharmaceutical industry [84].

## HCS AND TARGET VALIDATION

The target validation area of early drug discovery, and to a large extent all basic research, is focused on identifying new components in the cell biology puzzle and validating their various functions. On the basic research side, validation adds to the understanding of the big picture. On the drug discovery side, validation provides the foundation to develop assays that reflect disease states so that molecules that perturb the disease state can be identified. Ultimate success in this area requires both relevant biological models and physiologically accurate environmental conditions. The relatively recent technological advances of using stem cells and RNAi to create cell models are a natural fit for phenotypic quantitation. Whether it is tracking the development of a differentiating population of stem cells en route to becoming muscle cells, or assessing the outcome of knocking out proliferation signals for neurite outgrowth, HCS can be applied. In a recent review of RNA interference based screening, Perrimon comments, “Perhaps the most significant advances in RNAi HTS will come from high content screening. Cell-based HCS that rely on cellular phenotypes are becoming one of the preferred methods in RNAi HTS because they generate data sets that are rich in information…the use of primary cells offers ample opportunities to carry out cell morphology screens in a biologically relevant context” [85].

Moffat *et al*. developed a screen based on high content imaging to identify genes required for mitotic progression and applied it to 5000 unique short hairpin RNA (shRNA) expressing lentiviruses targeting 1028 human genes [86]. The screen identified ~ 100 (new) candidate regulators of proliferation. Similar studies using HCS to monitor phenotypic endpoints have been done using short interfering RNA (siRNA) libraries [87-90].

On the stem cell front, HCS has been used to help identify components of the regulatory machinery involved in stem cell self-renewal [91, 92] and differentiation [93], primarily through the quantitation of pluripotency markers like Oct-4 in either embryonic stem cells [94, 95] or lines derived from adult tissues [96]. Downstream tracking of cell fate using various differentiation state biomarkers has also been done [97]. The unique ability of HCS to provide spatial information about cell /cell relationships is illustrated by Peerani *et al*., where the heterogeneous microenvironments (niches) in the organization of human embryonic stem cell (hESC) cultures influence hESC fate [98]. By evaluating niche size and cell composition against localized secretion of differentiation inducing and inhibiting factors (*via* siRNA knock down) they discovered, “for the first time, a role for Smad1 in the integration of spatial information and in the niche-size-dependent control of hESC self-renewal and differentiation.”

## FUTURE DIRECTIONS AND OUTLOOK

It is clear from the published literature cited above that HCS has moved beyond the “proving the technology” phase and is now entering the “broad adoption” phase where the needs of an ever increasing user base will drive the technology to be more cost effective, easy to use, and robust. Development of new reagents [99] and micro-environments, combined with continued application of autofluorescent proteins [100] will open the door to even more ways to quantify cellular behavior. A focus on the productivity metrics of HCS, both in creating the information (i.e. assay prep automation, reagent kits, algorithms) and understanding it (i.e. data management, visualization, mining) will supplement the detection platforms, allowing more routine generation of HCS data as part of decision support in the life sciences. While the core applications of HCS will evolve to mass use, technology development will progress toward expanding biological data relevance through use of multicellular assemblies, tissues, organs, and organisms. An additional trend to incorporate “cellomics” with genomics and proteomics technologies will provide an unprecedented picture of cell functions.

The outlook for HCS is very bright, considering that many countries are proposing moratoriums to reduce animal testing [101, 102] in the “not-to-distant” future. The development of *in vitro* cell based assay tools like HCS could not be more timely. As someone who has participated in developing this technology from its inception, I can honestly say, due to the passion and dedication of “highly productive users”, HCS is taking its place as a powerful tool to help weave the “fabric of scientific knowledge” for years to come.

## Figures and Tables

**Table 1.** Key Application Areas of HCS and Example References

Application Area	Example	References
**Cell Signaling**	NFkB	20
	STAT	21,22
	Wnd/fzd	23
	akt	24
	NFAT	25,58
	P38	26
	TGF-beta	27,29
	Smad2/3	28,29
	GPCR	31-33,100
	Glucocorticoid receptor	15
	FOXO1a	58
	yeast	11
**Cell Physiology**	Proliferation	12,39-41,90
	Phosphorylation	13
	Phagocytosis	5
	Autophagy	6
	Gap junction induction	2
	Mitochondrial health	73,74,82-84
	Nuclear morphology	19,83,84,88
	Apoptosis	34-38,65
	Membrane permeability	82-84
	Cell cycle	19,42-44,58,86,99
	Motility/migration	46-51
	Cytoskeletal rearrangement	7,33,56,57
	Neurite outgrowth	9,14,59-65,72,80
	Transformation	45
***In Vitro* Toxicology**	Micronucleus induction	77,78
	Phospholipidosis	79
	Neurotoxicity	80
	Organelle health	73,74,82,84
	Hepatotoxicity	82-84
**Organism Physiology**	Cardiac failure	1
	Circadian rhythms	10
	Immunosupression	3
	Osteoporosis	4
	Fungal pathogenesis	8
	Virus neutralization	17
	Parasite infection	16
	Angiogenesis	54,55
	Alzheimer’s Disease	66
	Parkinson’s Disease	67,68
	Huntington’s Disease	69,70
	Amyotrophic Lateral Sclerosis	71
**Target Validation**	shRNA interference library	86
	siRNA interference library	87-90
	Stem cell self-renewal	91,92,98
	Stem cell differentiation	94-96,98
